# An atom efficient, single-source precursor route to plasmonic CuS nanocrystals†

**DOI:** 10.1039/c8na00325d

**Published:** 2018-11-06

**Authors:** Patrick Bergstrom Mann, Iain J. McGregor, Struan Bourke, Mary Burkitt-Gray, Simon Fairclough, Michelle T. Ma, Graeme Hogarth, Maya Thanou, Nicholas Long, Mark Green

**Affiliations:** Department of Physics, King's College London Strand London WC2R 2LS UK mark.a.green@kcl.ac.uk; Department of Imaging Sciences and Biomedical Engineering, King's College London, St Thomas' Hospital London SE1 7EH UK; Department of Chemistry, King's College London Britannia House London SE1 1DB UK; Institute of Pharmaceutical Sciences, King's College London Franklin Wilkins Building London SE1 9NH UK; Department of Chemistry, Imperial College London Kensington London SW7 2AZ UK

## Abstract

The synthesis of colloidal semiconductor nanocrystals (NCs) from single-source precursors offers simplified manufacturing processes at the cost of reduced atom efficiency. Self-capping routes have the potential to maximise this efficiency although investigation has so far been limited to organic solvents. Here we present the synthesis of copper sulfide NCs *via* the decomposition of a copper dithiocarbamate complex in water. Nanocrystalline covellite particles were prepared without the need for additional capping ligand and exhibited a hollow nanosphere morphology. Mass spectrometry of the water-stable NCs indicated the presence of a number of surface ligands, including a small amine fragment of the single-source precursor (SSP) complex. A broad plasmon resonance in the near-infrared (NIR) at 990 nm was also observed and the photothermal effect of this demonstrated. Cytotoxicity experiments indicated cell viability remained above 95% for NC concentrations up to 1 mg mL^−1^, indicating high biocompatibility.

## Abstract 2

The synthesis of colloidal semiconductor nanocrystals (NCs) from single-source precursors offers simplified manufacturing processes at the cost of reduced atom efficiency.

## Graphical abstract

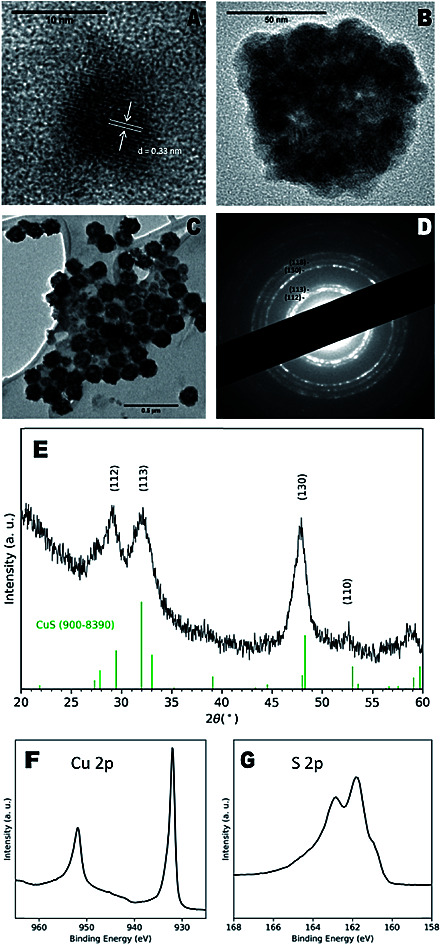

Single-source precursors (SSPs) offer facile routes to colloidal nanocrystals (NCs) through simplified procedures with fewer reagents.^1^ These precursors are metal complexes that undergo thermal decomposition to provide the constituent atoms for a given NC. SSPs have also proven to be useful for the chemical vapor deposition (CVD) of thin films, allowing for uniform precursor volatility and typically lower toxicity.^2^ Often however, synthetic simplicity comes at the cost of more complicated SSP preparation and lower atom efficiency through wastage of the precursor ligands. Common SSPs for the preparation of II–VI and I–III–VI semiconductor NCs include dithio- and diseleno–carbamate complexes for binary materials,^1,3,4^ and bimetallic complexes with triphenylphosphine and chalcogenolate ligands for ternary materials.^5,6^ Whilst these precursors do provide novel routes, their atom efficiency is particularly poor, for instance, 3.4% in the case of CuInS_2_ synthesised from (Ph_3_P)_2_CuIn(SPh)_4_.

Improvements in atom efficiency whilst retaining the benefit of simplified synthetic procedures led to the development of so-called ‘self-capping’ methods. This involves the thermal decomposition of a SSP in which all or part of the precursor ligand becomes the NC capping agent. One of the earliest examples of this is the preparation of silver NCs from silver myristate, stearate and oleate complexes.^7^ The long-chain fatty acids of the SSP bound to the surface of the NCs, providing colloidal stability in organic solvents. Later, Lazell and O'Brien described the preparation of dithiocarbamate SSPs for the self-capping synthesis of hydrophobic CdS and Bi_2_S_3_ NCs.^4^ Octadecylamine-derived dithiocarbamate ligands were used to complex Cd or Bi for the production of NCs stabilised by the long-chain amine upon decomposition. Similar long-chain alkylthiolate complexes of copper have been used in the solventless synthesis of Cu_2_S nanorods.^8^ Decomposition of these complexes in the absence of solvent resulted in monodisperse NC samples, with a range of morphologies that could be achieved through decomposition temperature control. A more recent example concerns the synthesis of metal oxide NCs from 2-ethylhexanoate complexes in *n*-butylether.^9^ Small (sub 3 nm), monodisperse NCs of both SnO_2_ and CeO_2_ were synthesised *via* this method, with the materials exhibiting quantum confinement.

The work presented here investigated the self-capping synthesis of NCs in water from a metal dithiocarbamate SSP. In particular, the preparation of plasmonic copper sulfide has been undertaken as this material has shown promise in a number of biomedical applications, utilising the near infrared (NIR) absorption.^10,11^ A concise review of plasmonic NCs is beyond the scope of this paper, however we refer the reader to a recent review on the topic.^12^

This is to the best of our knowledge, the first example of a self-capping NC synthesis in water. Synthesis in water of colloidal NCs is of particular interest for the development of biocompatible nanomaterials as no subsequent ligand exchange or encapsulation procedure is required. It was hypothesised that 2,2′-(dithiocarboxyazanediyl)diacetic acid (1, Scheme 1a) could be used as a precursor ligand that formed a capping agent upon decomposition. Previous work characterised the decomposition product of a similar dithiocarbamate ligand used in the synthesis of fluorescent CdS NCs.^13^ In that case, a water-soluble Cd dithiocarbamate complex was heated with guanosine triphosphate to give CdS NCs and a cyclic molecule formed from the dithiocarbamate ligand. A similar decomposition pathway was suggested for the synthesis of plasmonic copper sulfide from a copper dithiocarbamate SSP complex (Scheme 1b).

**Scheme 1:**
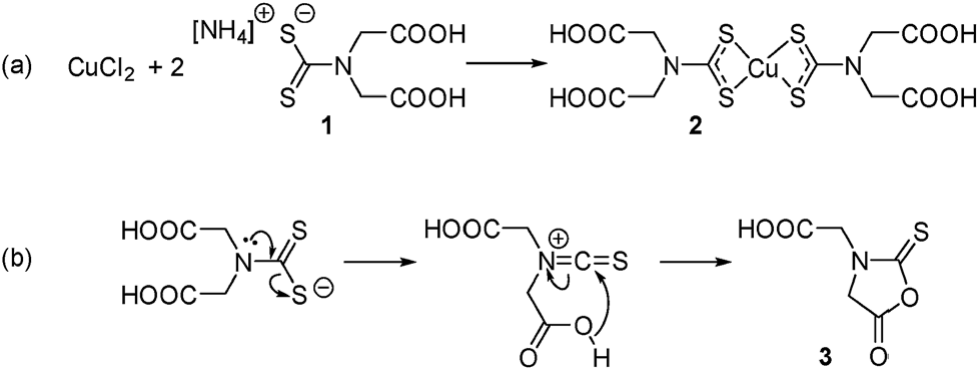
(a) Preparation of the water-soluble SPP and (b) the hypothesised thermal decomposition of the dithiocarbamate ligand.

For this investigation, copper(ii) bis-(2,2′-(dithiocarboxyazanediyl)diacetic acid) (2) was prepared and isolated as described previously.^14^ Electrospray ionisation mass spectrometry (ESI-MS) of an aqueous sample confirmed the formation of 2 (Fig. S1†). To synthesise NCs, 0.1 mmoles of this water-soluble copper dithiocarbamate complex in water (2 mL) was injected into 20 mL of water at 90 °C in a three-neck flask fitted with a condenser. Maintaining the temperature at 90 °C, the dark solution was heated with continuous stirring for 4 hours, with aliquots (1 mL) removed periodically. A color change from yellow to green was observed, signifying the growth of copper sulfide NCs. The NCs were isolated through the addition of an equal volume of 2-propanol and collected *via* centrifugation (4000 rpm for 5 minutes), before resuspension in water. Further washing steps were carried out with water in centrifuge filters (Vivaspin, 10 kDa MWCO) to remove excess ligand fragments (full experimental details are included in the ESI†).

Transmission electron microscopy (TEM) images of as-prepared particles indicated the presence of discrete NCs with a diameter of 8 ± 1 nm and clear lattice fringes (Fig. 1a), with a measured d-spacing of 0.33 nm, corresponding to the (110) plane of CuS. However, larger aggregate structures of multiple NCs were more prevalent (Fig. 1b) and exhibited diameters of 100 ± 40 nm. These larger particles also exhibit a hollow morphology (Fig. 1c) similar to CuS nanospheres reported elsewhere.^15,16^ Hollow structures can form due to the nanoscale Kirkendall effect in which the relative diffusion rates of constituent atoms result in an accumulation of vacancies in the centre of the NCs,^17^ however in this case, we suggest agglomeration of smaller particles may be the driving factor.

**Fig. 1:**
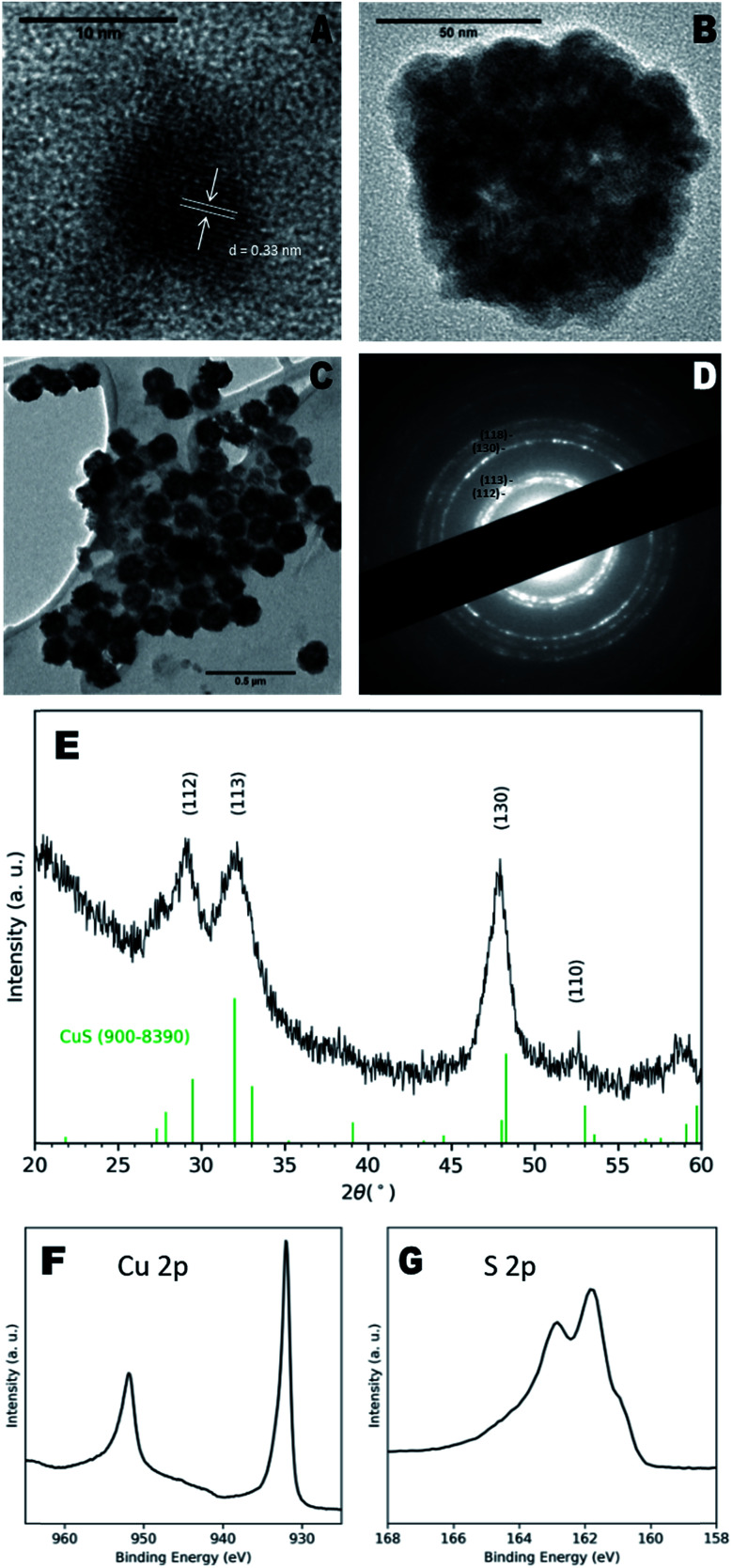
(A) TEM image of single CuS NC. (B) TEM image of a structure consisting of multiple aggregated CuS NCs. (C) The majority of particles observed are large structures with a hollow sphere morphology, as seen by TEM. (D) SAED pattern of as-prepared NCs, indicating a hexagonal crystal structure. (E) XRD pattern of NC sample indicates presence of covellite phase. (F) Cu 2p and (G) S 2p regions obtained from high resolution XPS characterisation.

Dynamic light scattering (DLS) measurements of NCs in water indicated a hydrodynamic diameter of 100 ± 30 nm and polydispersity index of 0.10 ± 0.03, confirming the presence mainly of the larger particles (Fig. S3†). The hypothesised capping ligands were small organic molecules that provide little steric hindrance for colloidal stability, likely leading to the aggregation observed here.

X-ray diffraction (XRD) analysis shown in Fig. 1e indicated a covellite (CuS) NC phase, characterised by broad peaks at 29.15°, 31.99°, 47.87° and 52.5° identified as the (112), (113), (130) and (110) planes respectively (ref. 900–8390), although the reflections appeared to have shifted to the left of the standard pattern, possible due to the sulfur-rich phase.^18^ The observed XRD pattern corresponded to the bands seen in the selected area electron diffraction (SAED) pattern (Fig. 1d). Energy-dispersive X-ray (EDX) spectroscopy of the samples reported a Cu : S ratio of 0.88 : 1 (Fig. S4†), close to the 1 : 1 ratio of the covellite phase, with the slight excess of S attributable to the S-containing species observed on the surface by ESI-MS, *vide infra*. X-ray photoelectron spectroscopy (XPS) spectra were recorded and quantitatively analysed, giving a Cu : S ratio of 0.82 : 1, which supports the EDX data. Cu 2p and S 2p peaks matched those previously ascribed to covellite,^19^ whilst a calculated auger parameter of 1850.3 eV also corresponded exactly to that of covellite.^20^

A surface plasmon resonance was observed in the absorption spectra of as-prepared copper sulfide in the NIR region (Fig. 2), typical of other CuS nanomaterials.^21^ The absorption at 430 nm is due to ligand–metal charge transfer (LMCT) in the precursor complex, as seen for other Cu dithiocarbamate complexes.^22^ This precursor peak decreased during the reaction as a broad plasmon resonance peak centered around 990 nm increased. The plasmonic origin of this absorption feature was confirmed through the measurement of absorption in different solvents, resulting in a red-shift of the absorption peak with increasing solvent refractive index (Fig. S5†). No photoluminescence was observed from these NCs.

**Fig. 2:**
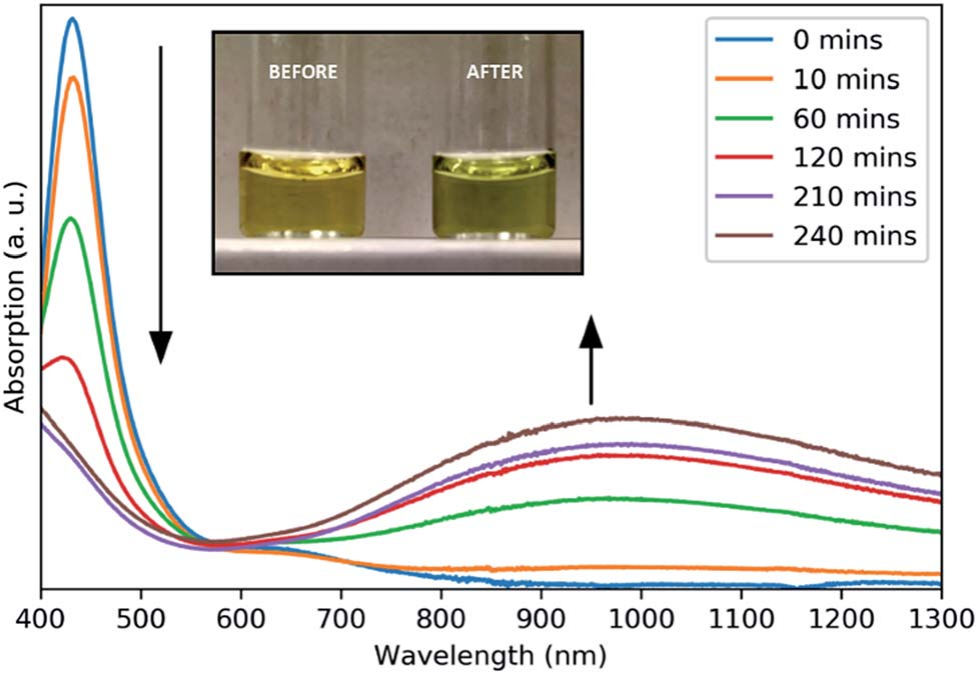
Absorbance spectra of aliquots taken at intervals throughout the growth of CuS NCs. Inset: a photograph of the reaction solution before and after heating.

ESI-MS was used to identify the ligand species present on the NC surface. Samples were analysed before and after cleaning to identify both decomposition products and capping ligands. Based on previous work,^13^ we expected the formation of 2-(5-oxo-2-thioxooxazolidin-3-yl)acetic acid (3, Scheme 1b), a five-membered ring with carboxylic acid functionality and an expected *m*/*z* ratio of 173.99 in negative mode ESI-MS (positive mode ESI-MS gave no useable data). There were no corresponding peaks observed in mass spectra taken before (Fig. S6†) or after (Fig. 3) NC cleaning, suggesting either the formation of the species doesn't occur or that it is rapidly hydrolysed under the reaction conditions.

**Fig. 3:**
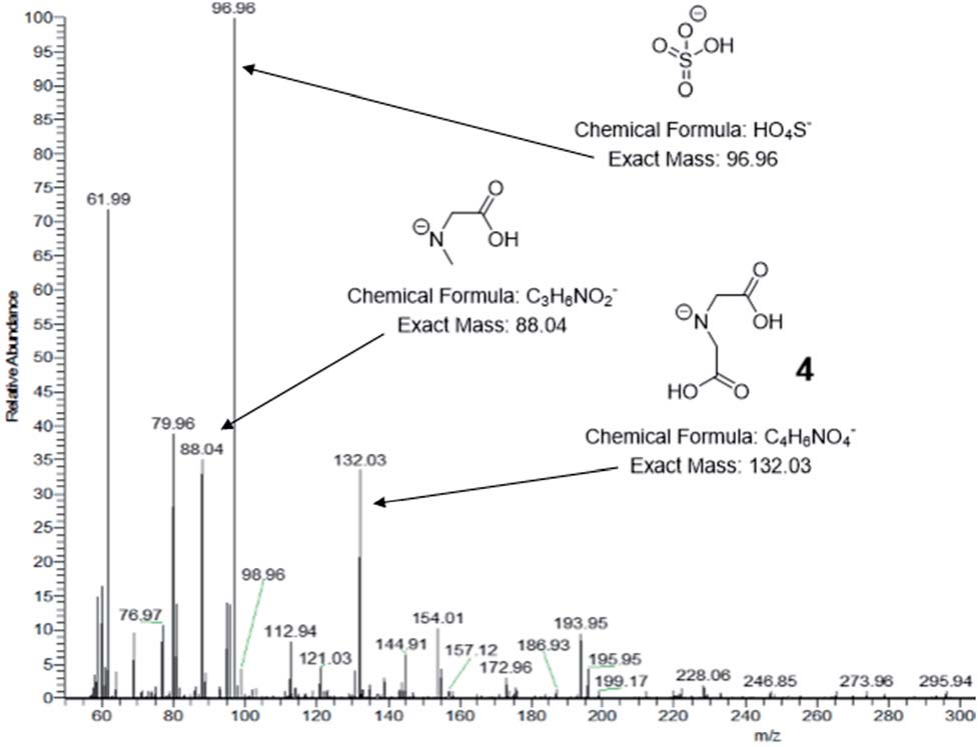
Negative mode ESI mass spectra of CuS NCs after isolation and centrifugal washing. Significant peaks are indicated along with their corresponding structures.

Post-washing ESI-MS (Fig. 3) indicated the presence of a species with an *m*/*z* ratio of 132.03, corresponding to the deprotonated amine, iminodiacetic acid (4). Its presence indicates cleavage of the C–N backbone bond in the dithiocarbamate ligand, giving a water-soluble amine capable of binding to the CuS clusters and providing colloidal stability. The sodium salt and a singly decarboxylated derivative of 4 are also observed at 154.01 and 88.04 respectively. A less abundant signal at 76.97 can be attributed to hydrogen monothiocarbonate, [CSO_2_H]^−^, which likely arises from the C of the C–N ligand backbone and one of the connected S atoms in a hydrolysis process. A dithiocarbonate signal is not observed suggesting that reaction occurs *via* an alkylisothiocyanate intermediate. It is unclear whether these species are formed *via* the proposed cyclic molecule but it is apparent that significant hydrolysis of the dithiocarbamate occurs.

Further S-containing species can be seen at *m*/*z* ratios of 96.96 and 112.94 corresponding to hydrogen sulfate, [HSO_4_]^−^, and hydrogen thiosulfate, [HS_2_O_3_]^−^. These species arise from the Cu : S ratio of 1 : 4 in the precursor complex, which results in a large excess of S atoms that are not incorporated into the CuS NCs. The S-containing species can be identified by XPS in the S 2p region, with binding energies higher than those of the metal sulfide, resulting in a shoulder around 165 eV. Again, this accounts for the excess of S over Cu as observed by EDS and XPS. A signal at 61.99 can be attributed to nitrate, [NO_3_]^−^, which further suggests complete hydrolysis of some of the dithiocarbamate ligands has occurred. A *ζ*-potential measurement of −8.0 ± 0.4 mV was recorded, consistent with an NC surface stabilized by negatively charged species, including the small inorganic anions and carboxylic acid-containing amine species.

In order to approximate a theoretical atom efficiency for this SSP it was assumed that decomposition occurs to give primarily, CuS, iminodiacetic acid, hydrogen sulfate and hydrogen monothiocarbonate. These species account for all parts of the SSP complex, with the addition of water and oxygen to balance the equation (Scheme S1†). Other species observed by ESI-MS were assumed to be further decomposition or hydrolysis products of these fragments as discussed above. Assuming all species are bound to the NC surface then an efficiency of 90.6% to 94.3% can be calculated, depending on protonation of the carboxylic acid groups (full details in ESI†). However, if only the iminodiacetic acid remains bound to the NC surface, then an efficiency of 60.4% to 64.2% is achieved.

In comparison to traditional SSPs, the atom efficiency is considerably higher even if not all of the resulting species remain bound. Copper(ii) diethyldithiocarbamate for example would result in an atom efficiency of 5.4–8.1% depending on whether CuS or Cu_2_S is formed. Improvements could be made to the SSP used here such that the extent of hydrolysis is controlled, most likely through the optimisation of reaction temperature and time.

To demonstrate their potential for biological use, synthesised nanoparticles were washed in a centrifugal filter and their cytotoxicity in HeLa cells assessed. Fig. 4a shows the minimal effect of the particles (1 mg mL^−1^) on cell structure and behaviour, indicating significant biocompatibility. Quantitative assessment using live/dead cell staining indicated only a 2% decrease in cell viability after 24 hours for a concentration of 1 mg per mL (Fig. 4b). To further demonstrate the therapeutic utility of these particles, their photothermal effect was measured using a 785 nm laser as the excitation source, with a power comparable to that used in other studies.^16,23^ An increase of 15.1 °C was observed for the CuS suspension over 3 minutes, whilst the water control exhibited an increase of only 0.7 °C. Higher temperature changes may be possible for lower nanoparticle concentrations if a higher wavelength laser is used (800–900 nm), at which the CuS absorption is greater (Fig. S8†), although this was not possible during the study presented here.

**Fig. 4:**
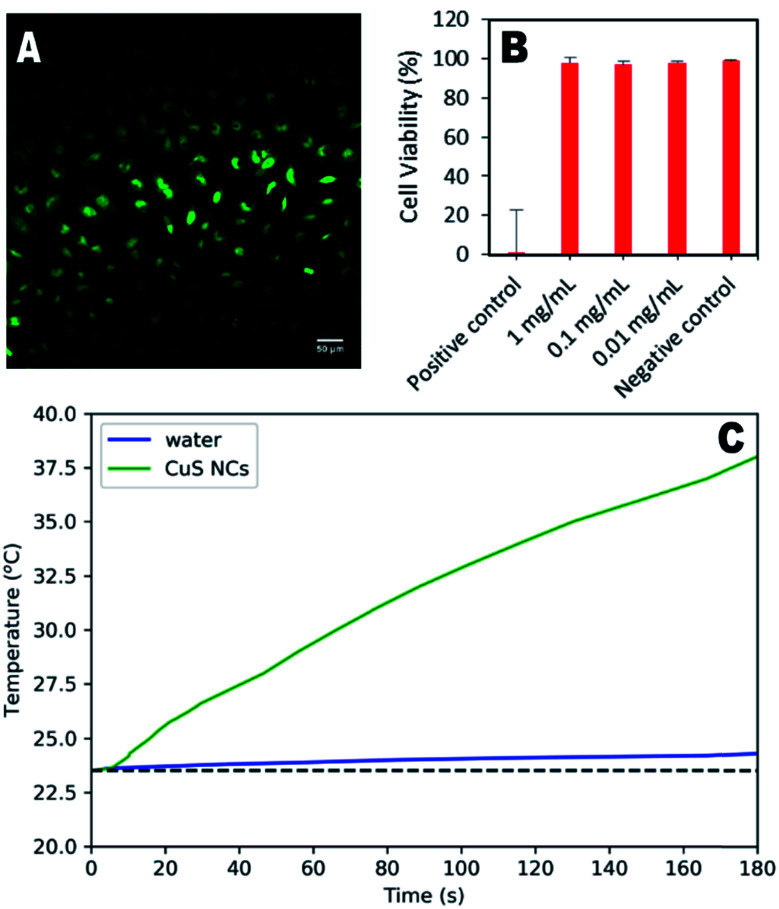
(A) Representative image of stained HeLa cells exposed to 1 mg mL^−1^ CuS NCs, imaged at 20× magnification. (B) Cell viability of HeLa cells incubated (37 °C, 5% CO_2_) with 1, 0.1 or 0.01 mg mL^−1^ CuS NCs for 24 hours, quantified by live/dead staining with Nuc488 green stain. (C) Photothermal effect of CuS NCs (20 mg mL^−1^, 500 μL) irradiated with a 785 nm pulsed laser (80 MHz, 3.0 W cm^−2^) compared to an ultrapure water control.

## Conclusions

In conclusion, CuS NCs have been synthesised in aqueous conditions from a single molecular precursor with atom efficiency up to 94%. Particle morphology suggests the agglomeration of smaller particles plays a part in NC growth although further work is required to elucidate the mechanism by which this occurs. Mass spectrometry indicated the decomposition of the ligand *via* cleavage of the C–N backbone bond to give an amine capping agent, as well as other hydrolysis products of the dithiocarbamate ligand. The amine-capped NCs were stable in water with an average hydrodynamic diameter of 97 nm and exhibited a broad plasmon resonance around 990 nm. Assessment *in vitro* found the particles did not significantly reduce the viability of HeLa cells and their potential use as a photothermal therapy agent has been demonstrated.

## Conflicts of interest

There are no conflicts to declare.

## Supplementary Material

NA-001-C8NA00325D-s001

## References

[cit1] Pickett N. L., O'Brien P. (2001). Chem. Rec..

[cit2] Knapp C. E., Carmalt C. J. (2016). Chem. Soc. Rev..

[cit3] Lazell M., Norager S. J., O'Brien P., Revaprasadu N. (2001). Mater. Sci. Eng., C.

[cit4] Lazell M., O'Brien P. (1999). Chem. Commun..

[cit5] Castro S. L., Bailey S. G., Raffaelle R. P., Banger K. K., Hepp A. F. (2003). Chem. Mater..

[cit6] Banger K. K., Jin M. H. C., Harris J. D., Fanwick P. E., Hepp A. F. (2003). Inorg. Chem..

[cit7] Abe K., Hanada T., Yoshida Y., Tanigaki N., Takiguchi H., Nagasawa H., Nakamoto M., Yamaguchi T., Yase K. (1998). Thin Solid Films.

[cit8] Sigman M. B., Ghezelbash A., Hanrath T., Saunders A. E., Lee F., Korgel B. A. (2003). J. Am. Chem. Soc..

[cit9] Kim Y. J., Kim Y. S., Chai S. Y., Cha D. H., Choi Y. S., Lee W. I. (2007). New J. Chem..

[cit10] Goel S., Chen F., Cai W. (2014). Small.

[cit11] Zhou M., Tian M., Li C. (2016). Bioconjugate Chem..

[cit12] Comin A., Manna L. (2014). Chem. Soc. Rev..

[cit13] Green M., Sandiford L., Anderson K. M., Ma Y. (2012). ChemPlusChem.

[cit14] Jones M. M., Burka L. T., Hunter M. E., Basinger M., Campo G., Weaver A. D. (1980). J. Inorg. Nucl. Chem..

[cit15] Leidinger P., Popescu R., Gerthsen D., Lünsdorf H., Feldmann C. (2011). Nanoscale.

[cit16] Ramadan S., Guo L., Li Y., Yan B., Lu W. (2012). Small.

[cit17] Yin Y., Rioux R. M., Erdonmez C. K., Hughes S., Somorjal G. A., Alivisatos A. P. (2004). Science.

[cit18] We thank a referee for this interpretation

[cit19] Xie Y., Riedinger A., Prato M., Casu A., Genovese A., Guardia P., Sottini S., Sangregorio C., Miszta K., Ghosh S., Pellegrino T., Manna L. (2013). J. Am. Chem. Soc..

[cit20] Biesinger M. C. (2017). Surf. Interface Anal..

[cit21] Lewis D. J., Deshmukh P., Tedstone A. A., Tuna F., O'Brien P. (2014). Chem. Commun..

[cit22] Van Der Stam W., Berends A. C., De Mello Donega C. (2016). ChemPhysChem.

[cit23] Wang S., Riedinger A., Li H., Fu C., Liu H., Li L., Liu T., Tan L., Barthel M. J., Pugliese G., De Donato F., Scotto D'Abbusco M., Meng X., Manna L., Meng H., Pellegrino T. (2015). ACS Nano.

