# Supplementary material for: An atom efficient, single-source precursor route to plasmonic CuS nanocrystals
Source: Nanoscale Adv. 2018 Nov 6;1(2):522–6. doi: 10.1039/c8na00325d (PMC9473250; doi:10.1039/c8na00325d)
Supplement: NA-001-C8NA00325D-s001 [file NA-001-C8NA00325D-s001.pdf]

Electronic Supplementary Material (ESI) for Nanoscale Advances.
This journal is © The Royal Society of Chemistry 2018

Electronic Supplementary Information

An Atom-Efficient, Single-Source Precursor Route to Plasmonic CuS Nanocrystals

*Patrick Bergstrom Mann,[a] Iain J. McGregor,[b] Struan Bourke,[a] Mary Burkitt-Gray,[a] Simon Fairclough,[a] Michelle T. Ma,[b] Graeme Hogarth,[c] Maya Thanou,[d] Nicholas Long[e] and Mark Green[a]**

[a] Department of Physics, Strand Campus, King's College London, London, WC2R 2LS.

[b] Division of Imaging Sciences and Biomedical Engineering, St. Thomas' Hospital, King's College London, London, SE1 7EH.

[c] Department of Chemistry, Britannia House, King's College London, London, SE1 1DB.

[d] Institute of Pharmaceutical Sciences, Franklin Wilkins Building, King's College London, London, SE1 9NH.

[e] Department of Chemistry, Imperial College London, Kensington, London, SW7 2AZ.

*Corresponding author: mark.a.green@kcl.ac.uk

Experimental Section

Materials & Characterization: Copper (II) chloride ($CuCl_2$), carbon disulphide (CS_2), iminodiacetic acid, ammonium hydroxide (NH_4OH), Dulbecco's phosphate buffered saline (PBS), Triton X-100 and formaldehyde were supplied by Sigma Aldrich. Methanol (HPLC grade) and 2-propanol (HPLC grade) were supplied by Fisher Chemicals. Dulbecco's Modified Eagle Medium (DMEM), foetal bovine serum (FBS), penicillin–streptomycin-L-glutamine solution, Trypsin/EDTA Solution (TE) and NucGreen Dead 488 ReadyProbes (Nuc488) were purchased from Thermofisher Scientific. Microplates (8 x square wells) were purchased from Thistle Scientific Ltd. HeLa cells were supplied internally within King's College London and verified as HeLa by STR profiling from Eurofins MWG. Ultrapure water (Milli-Q System) used throughout. Transmission electron microscopy (TEM) was carried out using a Joel 2100 microscope (acceleration voltage 200 kV) for standard resolution imaging and selected area electron diffraction (SAED). Energy-dispersive X-ray spectroscopy (EDS) measurements were taken on Oxford Instruments. Samples were deposited from water onto Ni grids with ultrathin carbon film (3 mm, 400 mesh) and left to evaporate in ambient conditions. Absorption spectra were recorded using a Hitachi U-4100 UV-visible-NIR spectrophotometer in a quartz cuvette (pathlength of 1 cm). DLS size and ζ-potential measurements were recorded on a Zetasizer Nano-S (Malvern) at 25°C with samples in a quartz cuvette (pathlength of 1 cm). ESI-MS of aqueous nanocrystal suspensions was carried out by the mass spectrometry facility at King's College London. XRD patterns were obtained at the Department of Chemistry XRD facility, University College London. A Kratos Axis Ultra DLD system was used to collect XPS spectra using monochromatic Al Kα X-ray source operating at 150 W (10 mA x 15 kV). Data was collected with pass energies of 80 eV for survey spectra, and 40 eV for the high-resolution scans with step sizes of 1 eV and 0.1 eV respectively. The system was operated in the Hybrid mode, using a combination of magnetic immersion and electrostatic lenses and acquired over an area approximately 300 x 700 μm^2. A magnetically confined charge compensation system was used to minimize charging of the sample surface, and all spectra were taken with a 90° take off angle. A base pressure of ~ $1x10^{-9}$ Torr was maintained during collection of the spectra. Data was analysed using CasaXPS (v2.3.19rev1.1l) after subtraction of a Shirley background and using modified Wagner sensitivity factors as supplied by the manufacturer.

Preparation of 2,2'-(dithiocarboxyazanediyl)diacetic acid (1): A solution of CS_2 (10 mL, 0.17 mol) in methanol (30 mL) was added to a cooled solution (0°C) of iminodiacetic acid (0.1 mol, 13.3 g) in methanol (70 mL) and aqueous NH_4OH solution (25%, 35 mL). The solution was reacted for 3 hours, after which the precipitate was collected and washed with copious amounts of methanol to remove unreacted iminodiacetic acid (10 x 50 mL). Yield = 14.3 g, 59%. ^{1}H NMR (400 MHz, D_2O, δ): 4.49 (broad singlet). ^{13}C NMR (100 MHz, D_2O, δ): 59.0, 176.4, 211.7. ESI-MS m/z: calcd for $[C_5H_6NS_2O_4]^-$ 207.9746; found 207.9744.

Preparation of copper (II) bis-(2,2'-(dithiocarboxyazanediyl)diacetic acid) (2): $CuCl_2$ (0.075 mmoles) was dissolved in methanol (1 mL) and added dropwise to a suspension of **1** (0.15 mmoles) in methanol (2 mL) with vigorous stirring. A dark green precipitate formed immediately. The solid was collected via filtration under gravity and washed with cold methanol to give **2** as a dark green powder. ESI-MS m/z: calcd for $[CuC_{10}H_{12}N_2S_4O_8]$ 478.8767; found 478.8759.

Nanocrystal Synthesis: In a typical procedure, **2** (0.1 mmol) was dissolved in water (2 mL) to give a dark yellow solution. This precursor solution was injected into water (20 mL) at 90°C and heating maintained for 4 hours. The reaction was cooled to room temperature and the larger material left to settle out. Nanocrystals were isolated through the addition of an equal volume of 2-propanol and collected via centrifugation (4000 rpm for 5 minutes), before resuspension in water. Dark green suspensions of CuS nanocrystals were washed thrice with ultrapure water in centrifuge filters (Vivaspin, 10kDa MWCO) to remove excess salts and ligand fragments not bound to the particle surface.

Atom efficiency of the nanocrystal synthesis was calculated as the percentage of starting material atoms that end up in the desired product (NC plus surface ligands). It was assumed only CuS, $[N(CH_2COOH)]^-$, $[SO_4H]^-$ and $[CSO_2H]^-$ are formed during decomposition, accounting for all parts of the SSP (Scheme S1). The atom efficiency will vary depending on the protonation state of the carboxylic acid groups and a range calculated from the case of both protonated and both deprotonated.

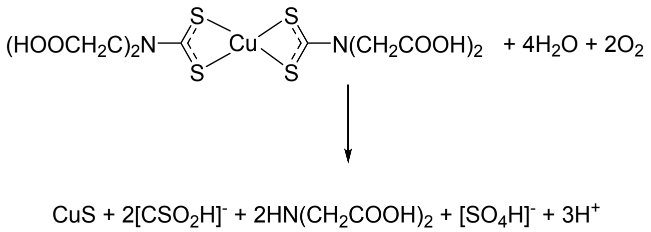

Scheme S1 A proposed equation for the decomposition of the SSP, balanced with water and oxygen for the calculation of an ideal atom efficiency.

Cell Viability: HeLa cells were grown on an 8-well μ-plate overnight at 30,000 cells ml^{-1}. The cells were incubated (37°C, 5% CO_2) with nanocrystal or control samples for 24 hours, before washing with PBS and incubating with Nuc488 for 15 minutes at room temperature in the absence of light. The cells were washed twice with PBS at room temperature and fixed with 4% paraformaldehyde (in PBS) for 20 min at room temperature. Cells were permeabilized by incubation (37°C, 5% CO_2) with 0.3% Triton X-100 in blocking buffer (0.5% bovine serum albumin, 0.1% NaN_3 in PBS) for 5 minutes followed by 3 x 5 minute incubations (37°C, 5% CO_2) in blocking buffer and 20 mM glycine. Cell media and oxidative nanoparticles known to be toxic used as negative and positive controls respectively. Fixed cells were imaged on an inverted Nikon Eclipse microscope using wide-field epifluorescence (Ti-E) equipped with a Cool SNAP HQ 2, DS-Fi2 Color CCD camera and 20x air objective. Original data were processed using NIS elements software (Nikon) and ImageJ (version no. 1.51j8 http://imagej.nih.gov/ij/, 1997–2018). The number of stained (dead) cells and total number of cells were counted manually in ImageJ. Cell viability has been reported as the percentage of unstained cells ± one standard deviation (n = 3).

Photothermal Measurements: CuS nanoparticles in water (20 mg ml^{-1}, 500 μL) were added to a quartz cuvette and placed in the path of an unfocused, pulsed 785 nm laser (80 MHz, 3.0 W cm^{-2}). The change in temperature was measured using a Pt/Ni type-K thermocouple and temperature logger over 3 minutes after start of laser irradiation. Water (500 μL) was used as a control.

Results & Discussion

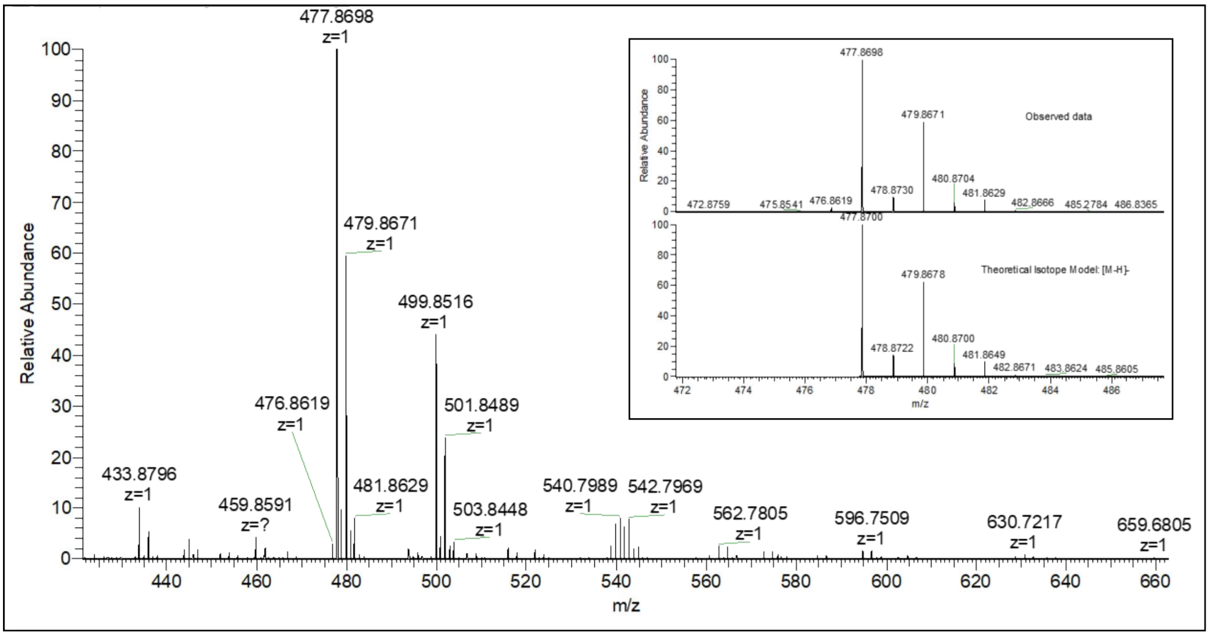

Figure S1 The negative mode ESI mass spectrum of **1**, exhibiting the expected complex, $[CuC_{10}H_{11}N_2O_8S_4]^-$, at a m/z ratio of 477.87. Inset: theoretical and observed isotope distributions for the expected complex ion $[CuC_{10}H_{11}N_2O_8S_4]^-$.

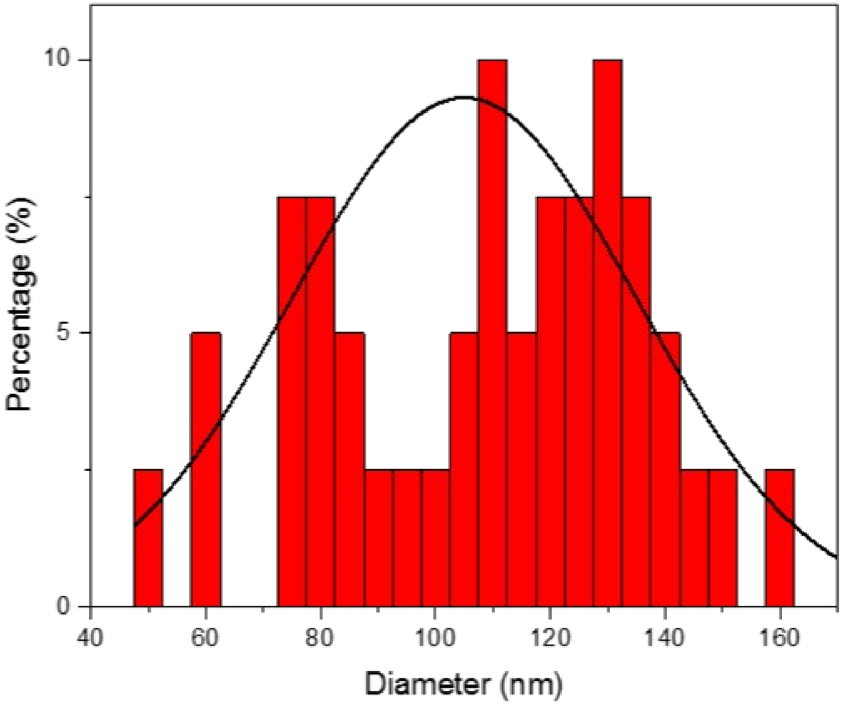

Figure S2 A histogram of diameters of the aggregate structures as measured from TEM micrographs using ImageJ (n=40). Size distribution is erratic, reflecting a low degree of control over synthesis using this single precursor.

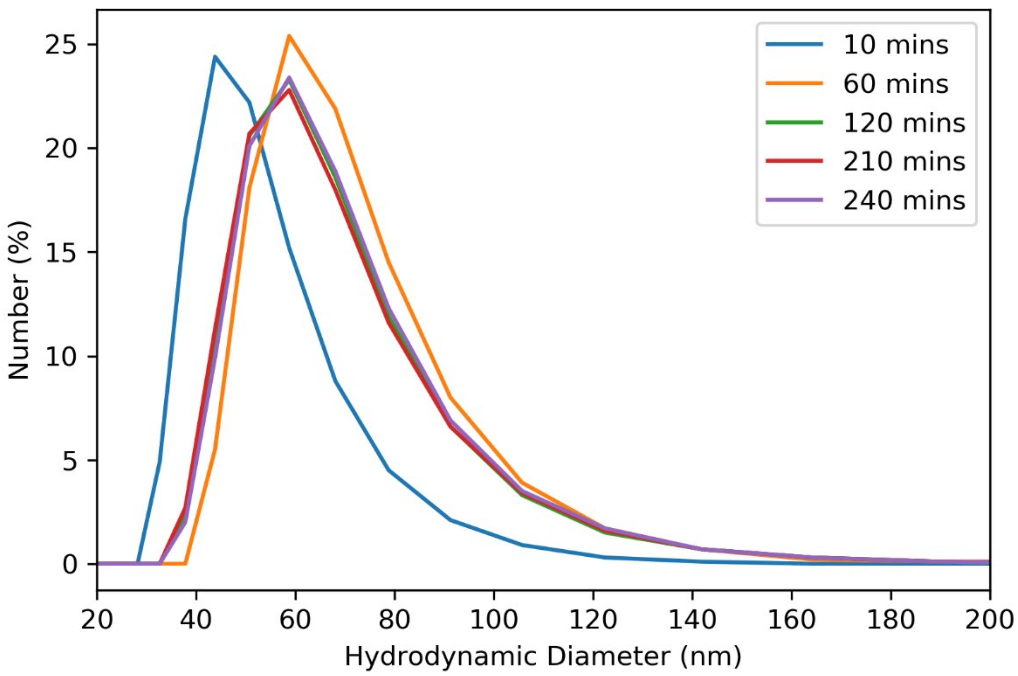

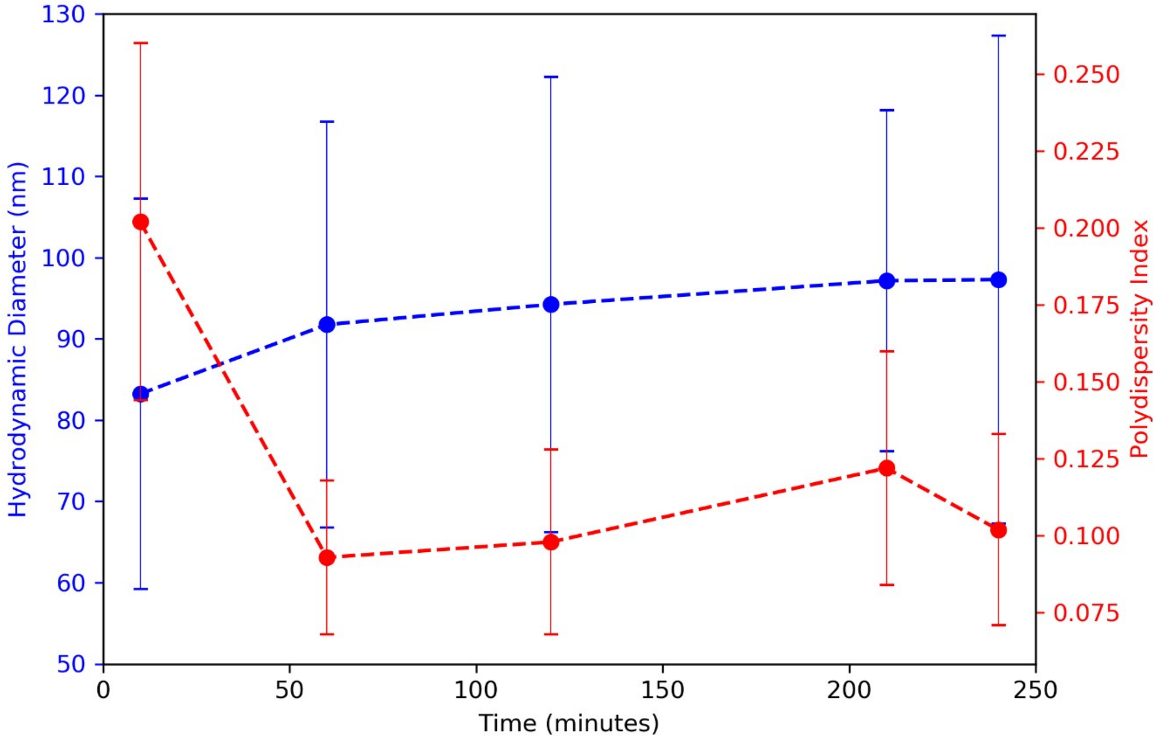

Figure S3 Top: Nanocrystal hydrodynamic diameter distributions as measured by DLS throughout their synthesis. Bottom: Hydrodynamic diameter (blue) and polydispersity index (red) values for aliquots of CuS nanocrystal suspensions taken periodically during synthesis.

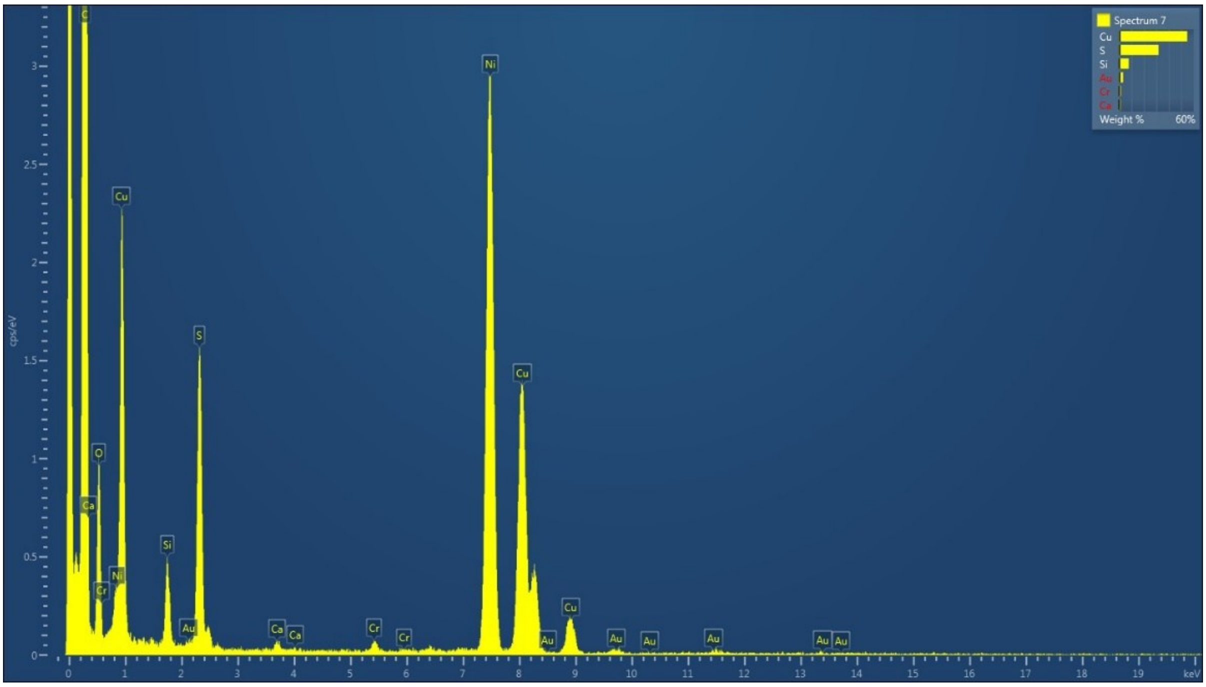

Figure S4 Energy-Dispersive X-Ray (EDX) spectrum for CuS nanocrystals supported on a Ni grid.

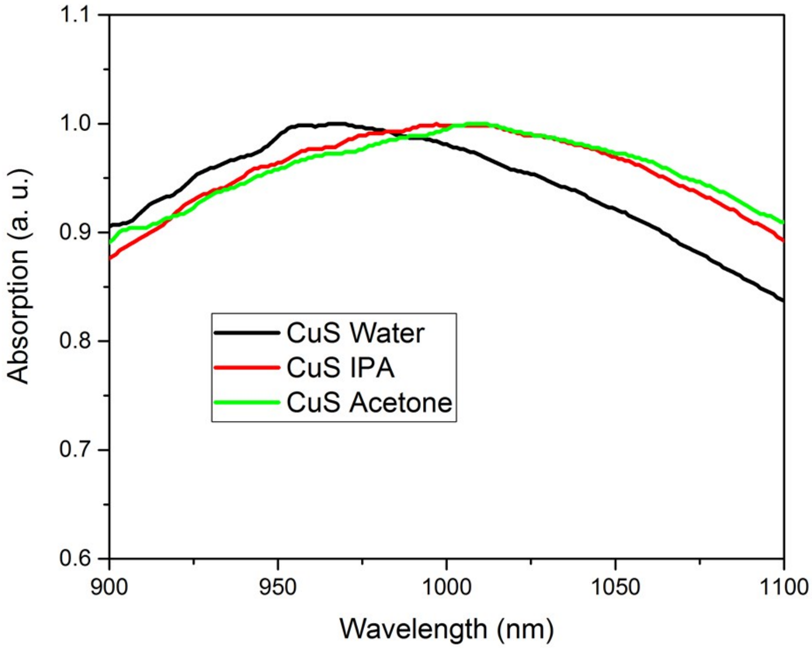

Figure S5 Absorption spectra of CuS nanocrystals suspended in water, isopropyl alcohol (IPA) and acetone, indicating a shift in absorption band with changing solvent refractive index.

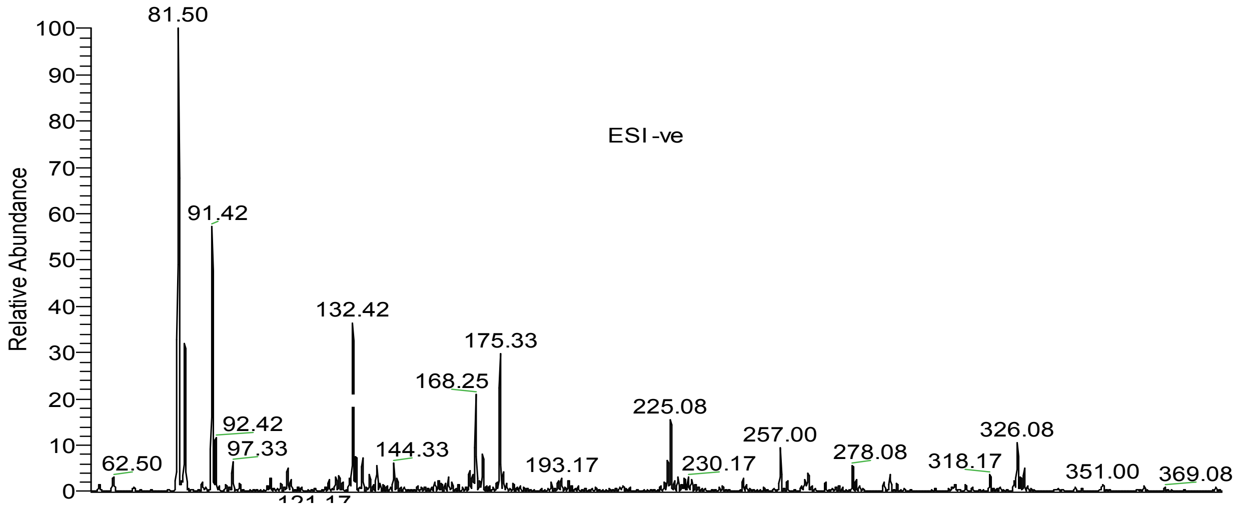

Figure S6 Negative Mode ESI mass spectrum of the crude CuS nanocrystal reaction solution after 4 hours and prior to any washing steps. None of the observed peaks could be attributed to expected fragments or the proposed cyclic decomposition product.

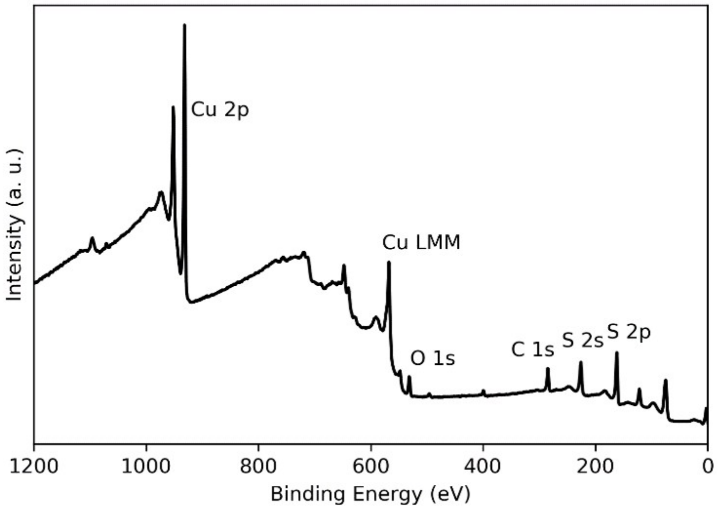

Figure S7 Wide field XPS scan of CuS nanocrystals with labelled regions of interest.

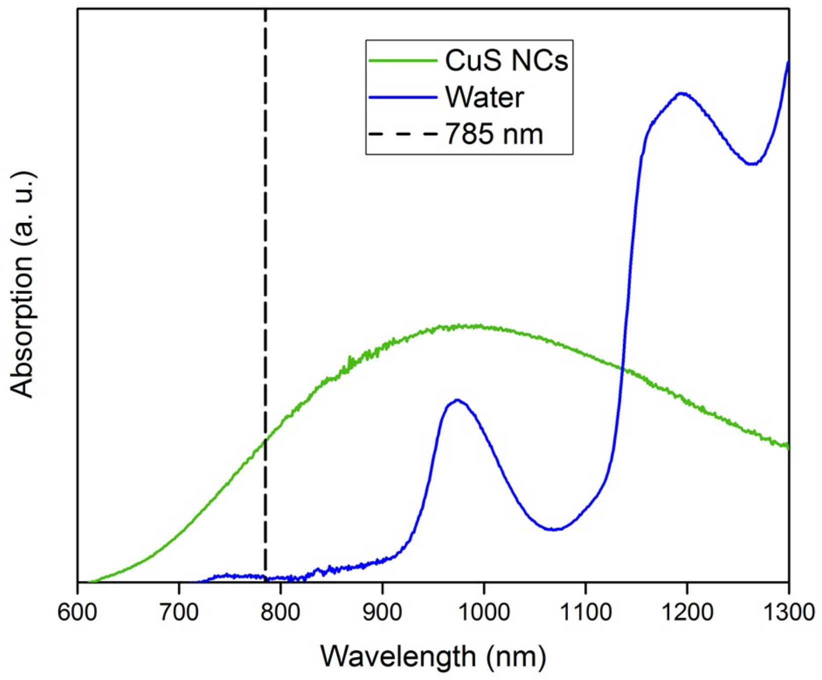

Figure S8 Spectrum showing the absorption of water and CuS nanocrystals, with 785 nm marked to indicate the laser wavelength used for photothermal measurements. The absorption by CuS is significantly greater than water at this wavelength although closer to 900 nm would allow for greater temperature changes and lower CuS concentrations. More appropriate wavelengths at the required power density were unavailable during this study.
